# Gastrocnemius Recession in Recalcitrant Plantar Fasciitis: A Systematic Review and Meta-Analysis of Randomized Controlled Trials

**DOI:** 10.3390/jcm15020616

**Published:** 2026-01-12

**Authors:** Arantxa Pérez González, Amadeo Sanz-Perez, Simone Moroni, Cristina Razzano, Juan Vicente-Mampel, Javier Ferrer-Torregrosa

**Affiliations:** 1 Podiatry Department, Faculty of Medicine and Health Sciences, Valencia Catholic University “San Vicente Mártir”, 46001 Valencia, Spain; arantxa.pg@mail.ucv.es (A.P.G.); javier.ferrer@ucv.es (J.F.-T.); 2 Departamento de Farmacología Farmacognosia y Botánica, Facultad de Farmacia, Universidad Complutense de Madrid, 28040 Madrid, Spain; amadeosa@ucm.es; 3 Doctorate School, Valencia Catholic University “San Vicente Mártir”, 46001 Valencia, Spain; simone.moroni@mail.ucv.es (S.M.); cristina.razzano@mail.ucv.es (C.R.); 4 Department of Physiotherapy, School of Medicine and Health Science, Catholic University of Valencia, Torrent, 46001 Valencia, Spain

**Keywords:** plantar fasciitis, chronic plantar fasciitis, gastrocnemius recession, medial gastrocnemius recession, gastrocnemius lengthening

## Abstract

**Background**: Recalcitrant plantar fasciitis (RPF) is a common cause of chronic heel pain, resistant to conservative treatment in up to 10% of cases. A biomechanical association exists between isolated gastrocnemius contracture and increased tension on the plantar fascia. In this context, gastrocnemius recession (GR) has emerged as a surgical intervention aimed at reducing fascial strain and improving functional outcomes. **Methods:** A systematic review and meta-analysis were conducted in accordance with PRISMA guidelines and registered with PROSPERO (CRD420251028862). Randomized controlled trials evaluating the efficacy of GR in patients with RPF were included. Searches were performed in PubMed, Cochrane Library, and Web of Science. Risk of bias was assessed using the RoB 2 tool, and the certainty of evidence was evaluated using the GRADE approach. Primary outcomes included foot function (AOFAS), pain (VAS), and ankle dorsiflexion range (FDTPA). **Results**: Five studies encompassing 150 patients were included. Meta-analysis revealed statistically significant improvements in foot function (AOFAS, standardized mean difference [SMD] = 0.81; 95% CI: 0.26–1.36), pain reduction (VAS, SMD = −1.17; 95% CI: −1.99 to −0.36), and ankle dorsiflexion (FDTPA, SMD = 0.74; 95% CI: 0.26–1.22). GR demonstrated advantages over plantar fasciotomy in terms of postoperative recovery and preservation of fascial structure. No major complications were reported, and some studies documented sustained benefits up to six years postoperatively. Moderate to high heterogeneity was observed, largely due to variations in surgical technique and follow-up durations. **Conclusions**: Gastrocnemius recession is a safe and effective surgical option for treating RPF, particularly in patients with isolated gastrocnemius contracture and failure of conservative therapies. It significantly improves foot function, reduces pain, and enhances ankle mobility, with durable long-term outcomes. Trial Registration: This systematic review is registered with PROSPERO (CRD420251028862).

## 1. Introduction

Heel pain is clinically characterized as discomfort localized primarily to the medial calcaneal tubercle. Its etiology is diverse, encompassing musculoskeletal, neurological, inflammatory, infectious, or systemic origins [1,2,3]. Clinically, plantar fasciitis, a term used broadly to describe both acute inflammatory and chronic degenerative conditions of the plantar fascia, typically presents as pain upon the first steps after rest [4,5]. It most commonly affects adults in their third to fifth decades of life, with an estimated prevalence ranging from 0.5% to 8% [6,7]. Although most cases respond to conservative treatment, approximately 5% to 10% progress to recalcitrant plantar fasciitis (RPF), defined as persistent symptoms despite adequate non-surgical management [8].

The plantar fascia (PF) is a dense connective aponeurosis primarily composed of type I collagen, anatomically organized into three bands—medial, central, and lateral. Its main function is to support the longitudinal arch and to contribute to the biomechanics of gait [4]. The PF exhibits essential stiffness required for effective transmission of forces generated by the triceps surae via the Achilles tendon [9]. Anatomically and biomechanically, there is a close relationship between the Achilles tendon and the plantar fascia, supporting the hypothesis that gastrocnemius dysfunction can directly impact the PF [10]. This interaction is best exemplified by the “windlass mechanism,” in which tension in the fascia during the propulsive phase of gait increases the rigidity of the medial arch, enabling the foot to act as a rigid lever for forward propulsion [5,11].

The posterior compartment of the lower limb plays a key role in this dynamic. When the gastrocnemius muscle is shortened, hypertonic, or functionally contracted, ankle dorsiflexion range of motion (ROM) is limited. This limitation can lead to excessive stress and posterior traction on the PF, resulting in chronic pain and dysfunction [12,13]. Multiple factors may contribute to this condition, including evolutionary adaptations, muscular changes, genetics, and sedentary lifestyle [12,14]. In this context, treatment of gastrocnemius contracture is not only viewed as a definitive intervention but also as a preventive strategy for various foot pathologies [14]. Contracture may substantially contribute to pain perception and prolong symptom duration in affected individuals [12,13].

RPF represents a significant therapeutic challenge, with considerable functional, social, and economic implications [4,6,15]. While most patients respond to non-operative treatments, a subset (5–10%) remains symptomatic beyond 6 to 12 months [15]. For these patients, surgical intervention becomes a reasonable consideration. Gastrocnemius recession (GR) has gained traction due to its strong biomechanical rationale and promising clinical outcomes [12,16]. Various surgical techniques have been developed to release or lengthen the gastrocnemius, including those described by Silfverskiöld, Baumann [17,18,19,20], Strayer [21,22], Vulpius, and Baker. These methods differ in the level of muscle-tendon release: more proximal approaches tend to offer greater stability, whereas more distal techniques may yield greater gains in dorsiflexion [8,23]. A comparative overview of the most widely used surgical techniques is summarized in Table 1.

Over time, surgical approaches for gastrocnemius recession have evolved from traditional open techniques to minimally invasive procedures guided by ultrasound [24,25]. In these modern methods, skin incisions are significantly smaller, and fascial release is performed under ultrasound visualization to enhance precision and minimize soft tissue damage [26]. While promising outcomes have been reported in terms of pain relief and functional improvement, the literature still reflects variability in patient satisfaction levels [27].

In this context, GR emerges as a promising surgical alternative for patients with RPF. By addressing underlying equinus deformity of muscular origin, GR facilitates reduction in plantar fascial tension. The present systematic review and meta-analysis aims to evaluate the efficacy and safety of GR, and to compare it with other surgical techniques, thereby contributing evidence-based insights to guide clinical decision-making in foot and ankle surgery.

## 2. Materials and Methods

### 2.1. Protocol Registration and Methodological Guidelines

This systematic review and meta-analysis was conducted in accordance with the Preferred Reporting Items for Systematic Reviews and Meta-Analyses (PRISMA) guidelines [28], which provide a standardized methodological framework to ensure transparency and reproducibility. The PRISMA checklist is detailed in Supplementary File S1. In addition, further methodological recommendations from the Prisma in Exercise, Rehabilitation, Sport medicine and SporTs science (PERSiST) guidelines [29] were also considered. The review protocol was prospectively registered in the PROSPERO database under ID number CRD420251028862.

### 2.2. Eligibility Criteria

Inclusion Criteria. Studies were eligible for inclusion if they met the following criteria: (i) articles describing surgical gastrocnemius lengthening and its outcomes in patients with recalcitrant plantar fasciitis (RPF); (ii) randomized controlled trials (RCTs) only; (iii) publications indexed in scientific databases; and (iv) studies conducted in human subjects. The review was deliberately restricted to RCTs in order to maximize internal validity and minimize selection and confounding biases.

Exclusion Criteria. The following exclusion criteria were applied: (i) publications not meeting the above standards; (ii) articles not describing gastrocnemius surgical treatment; (iii) articles not addressing plantar fasciitis as the target condition; and (iv) secondary studies, special articles or collaborative papers, letters to the editor, non-original guidelines/documents, and non-randomized observational studies or case series, which were excluded after screening and were not considered for a separate qualitative synthesis.

### 2.3. Research Question (PICO Framework)

To guide the literature search and establish eligibility criteria, the following PICO question was defined:

P (Population): Patients with recalcitrant plantar fasciitis.

I (Intervention): Surgical gastrocnemius recession using techniques such as Barouk, Baumann, Vulpius, Strayer, or Baker.

C (Comparison): Conservative treatment or other surgical interventions such as plantar fasciotomy.

O (Outcomes): Objective outcome measures such as the American Orthopaedic Foot & Ankle Society (AOFAS) score, Visual Analog Scale (VAS) for pain, Foot Dorsiflexion Test with Knee in Extension (FDTPA), Manchester Oxford Foot Questionnaire (MOxFQ), Foot and Ankle Ability Measure (FAAM), Foot Function Index. (FFI), and quality of life (SF-36).

### 2.4. Search Strategy

The literature search was conducted in the following electronic databases: PubMed/MEDLINE, Cochrane Library, and Web of Science. Controlled vocabulary terms and keywords were combined using Boolean operators (AND/OR), derived from the PICO question. A search equation was designed using keywords defined according to their specific descriptors in health sciences (DeCS) and Boolean operators such as “AND” and “OR”. The keywords used were as follows: “gastrocnemius recession”; “gastrocnemius release”; “gastrocnemius lengthening”; “gastrocnemius slide”; “Strayer procedure”; “plantar fasciitis”; “plantar heel pain”; “heel spur syndrome”; “chronic plantar fasciopathy”.

Filters applied included study type (clinical trials or case series). No publication date restrictions were applied due to the limited amount of specific literature. Search strategies for other databases were adapted using the Polyglot Search Translator Tool [30]. These full search strings are presented in Appendix A.

### 2.5. Data Extraction

Data extraction was performed independently by two investigators (APG and ASP), who applied a standardized protocol to minimize collection errors and bias. To organize the information, a spreadsheet was developed using Microsoft Excel(Version 16.105), where the main characteristics of each primary study were recorded. Extracted data included: author and year of publication, sample size (*n*), study design, intervention, comparison, and study outcomes (see Table 2).

One researcher (JVM) double-checked the included studies in the systematic search and refined the eligibility criteria to narrow the scope of the review. All discrepancies between reviewers were resolved through discussion and, when necessary, with the involvement of a third investigator (JFT). Inter-rater reliability criteria were applied to ensure consistency in the data collected, in accordance with methodological recommendations for systematic reviews.

### 2.6. Methodological Quality and Risk of Bias

The quality of the included studies was evaluated using two complementary approaches. First, the RoB 2 tool (Risk of Bias in Randomized Trials) proposed by the Cochrane Collaboration was applied, specifically designed to assess the risk of bias in randomized controlled trials. This instrument examines five key domains of study design and execution: the randomization process, deviations from intended interventions, incomplete outcome data, outcome measurement, and selection of the reported results. Each item was evaluated individually using the standardized categories “Yes” (low risk), “Unclear” (uncertain risk), or “No” (high risk). Judgments were summarized in a color-coded table: green for “Yes” (low risk), yellow for “Unclear” (uncertain risk), and red for “No” (high risk).

Second, the Grading of Recommendations, Assessment, Development and Evaluation (GRADE) system was used to rate the certainty of the evidence regarding the main outcomes. This system considers key factors such as risk of bias, inconsistency across studies, imprecision of estimated effects, and potential publication bias. The quality of the evidence was classified as high, moderate, low, or very low.

### 2.7. Statistical Analysis

This systematic review with meta-analysis was conducted following PRISMA guidelines and was prospectively registered in PROSPERO. The statistical analysis was quantitative and inferential, based on the synthesis of data extracted from randomized controlled trials that met strict eligibility criteria. Forest plots were used to assess the intervention effects, and Egger’s test was applied to detect publication bias. All analyses were performed using STATA v16.0 All analyses were performed using Stata Statistical Software, version 16 (StataCorp LLC, College Station, TX, USA).

## 3. Results

### 3.1. Included Studies

**Figure 1:**
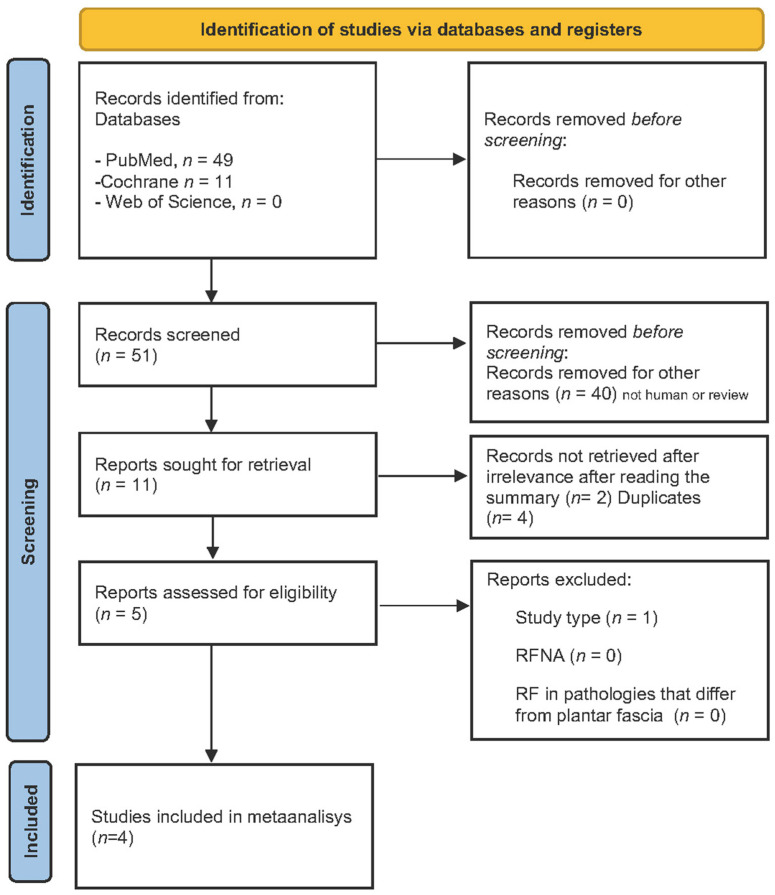
PRISMA Flow Diagram.

Following the search strategy and PICO-based selection criteria, 40 records were initially identified in PubMed, 11 in the Cochrane Library, and a smaller number (with no relevant studies) in Web of Science. After screening, duplicate removal, and critical appraisal, a total of five studies were included in the quantitative analysis. The complete study identification and selection process is summarized in the corresponding PRISMA flow diagram (Figure 1).

### 3.2. Study Characteristics

The included studies were published between 2018 and 2023 and were conducted in Norway [12,15] and Spain [16,27]. All selected studies evaluated the effect of GR as a therapeutic intervention in patients with RPF. Some studies compared this technique with alternative surgical procedures, such as open plantar fasciotomy (OPF), while others evaluated it against conservative treatment with stretching exercises.

The total aggregated sample comprised 150 patients, with individual study sizes ranging from 4 to 40 participants. Postoperative follow-up duration ranged from 3 months to 6 years. The general characteristics of each study, along with the techniques employed and the main outcomes, are summarized in Table 1.

#### Description of Surgical Interventions in the Included Studies

Across the randomized controlled trials included in this review, all primary surgical interventions were performed using open techniques, although some authors referenced the historical development of endoscopic alternatives. In Molund et al. (2018) [12], the intervention consisted of a proximal medial gastrocnemius recession (PMGR) following the technique described by Barouk. The procedure was undertaken through an open approach using a 3 cm transverse incision in the popliteal fossa, allowing direct identification and sectioning of the aponeurotic sheath of the medial gastrocnemius.

In the studies by Gamba et al. (2019a/2019b) [16,27], two distinct surgical techniques were compared, both executed through open approaches. The first was a proximal medial gastrocnemius release (PMGR) performed via a 3–4 cm incision just inferior to the popliteal crease, enabling controlled exposure of the musculotendinous junction. The second was an open partial plantar fasciotomy (OPF), carried out through a 3 cm medial incision at the calcaneal insertion of the plantar fascia, with resection limited to the medial third of the fascial band. Although the authors noted that endoscopic plantar fasciotomy is widely used in clinical practice, the study deliberately employed the open technique to ensure methodological comparability with the gastrocnemius release procedure.

The long-term follow-up study by Riiser et al. (2024) [15] applied the same PMGR technique described by Barouk, consistent with the original Molund trial, using an open approach with an incision of approximately 4 cm slightly distal to the popliteal fossa.

Overall, the three studies favored open surgical exposure to obtain direct visualization of the anatomical structures involved, whether the medial gastrocnemius aponeurosis or the plantar fascia, thereby facilitating a precise and complete section of the targeted fibers while minimizing the risk of injury to adjacent neurovascular structures.

### 3.3. Risk of Bias

This graphical representation provides a clear overview of the overall methodological quality of the included studies, facilitating comparison between them and highlighting the critical domains that may compromise the validity of the results (Table 3).

The GRADE system synthesizes the domains assessed for each study and the corresponding final quality of evidence, using an evidence profile table based on the key outcomes. This classification explicitly evaluates the confidence in the findings and facilitates the formulation of robust clinical recommendations (see Table 4).

The overall quality of the evidence was classified as high in studies that demonstrated a low risk of bias and adequate precision of the estimates, such as Molund et al., 2018 [12] and Riiser et al., 2023 [15], in which the methodological rigor, sample characteristics, and consistency of the findings contributed to a high level of confidence in the results. In contrast, although the studies by In Gamba et al., 2019a [16] and Gamba et al., 2019b [27]) were also clinical trials, the presence of methodological limitations related to risk of bias—mainly associated with partial blinding and potential uncontrolled confounding variables—together with a degree of imprecision attributable to sample size and variability in the observed effects, led to downgrading the quality of evidence to moderate. Accordingly, while these studies provide relevant data for the interpretation of the findings, their conclusions should be considered with greater caution when integrating results and formulating recommendations. This evaluation provides a transparent and structured basis to support future clinical recommendations for the surgical treatment of recalcitrant plantar fasciitis.

#### Quantitative Synthesis and Meta-Analysis

**Figure 2:**
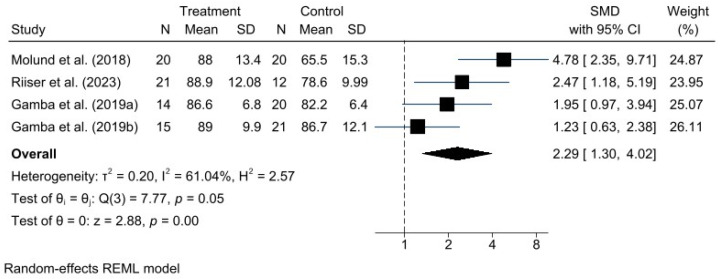
Forest plot for American Orthopaedic Foot & Ankle Society (AOFAS) scores after gastrocnemius recession procedure. *n* = total sample size, MD = mean differences, 95%CI = 95% confident interval. Studies included: Molund et al., 2018 [12]; Riiser et al., 2023 [15]; Gamba et al., 2019a [16]; Gamba et al., 2019b [27].

**Figure 3:**
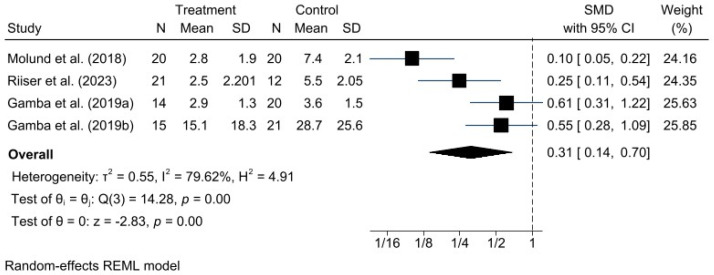
Forest plot for VAS pain scores after intervention. *n* = total sample size, SMD = standardized mean difference, 95% CI = 95% confidence interval. Studies included: Molund et al., 2018 [12]; Riiser et al., 2023 [15]; Gamba et al., 2019a [16]; Gamba et al., 2019b [27].

**Figure 4:**
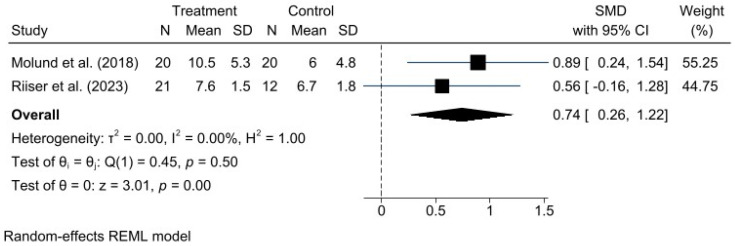
Forest plot for dorsiflexion range of motion (FDTPA) after intervention. *n* = total sample size, 95% CI = 95% confidence interval. Studies included: Molund et al., 2018 [12]; Riiser et al., 2023 [15].

A meta-analysis was performed for each clinical outcome using random-effects models. Results were organized into three main categories: functionality (AOFAS) (Figure 2), pain intensity (VAS) (Figure 3), and biomechanical improvement in dorsiflexion (FDTPA) (Figure 4).

### 3.4. AOFAS Scale

For functionality, measured with the AOFAS scale, the pooled analysis demonstrated a favorable effect of the GR technique, with a standardized mean difference (SMD) of 0.81 (95% CI: 0.26 to 1.36). Heterogeneity was moderate (I^2^ = 61.18%, H^2^ = 2.58), indicating some variability among studies, although not sufficient to undermine the robustness of the overall conclusion.

### 3.5. VAS for Pain

Regarding pain intensity, assessed using the VAS, a significant positive effect was also observed in favor of GR, with a standardized mean difference (SMD) of −1.17 (95% CI: −1.99 to −0.36). In this case, heterogeneity was higher (I^2^ = 79.62%, H^2^ = 4.91), suggesting greater variability among individual study results; however, this did not compromise the direction or validity of the estimated effect.

### 3.6. Foot Dorsiflexion Test with Knee in Extension

Regarding dorsiflexion, assessed using the FDTPA indicator, the pooled estimate revealed a significant improvement, with an SMD of 0.74 (95% CI: 0.26 to 1.22). Unlike the previous outcomes, no heterogeneity was detected (I^2^ = 0.00%, H^2^ = 1.00), indicating high consistency among the included studies. This absence of variability suggests that the results are primarily attributable to the intervention effect rather than chance, thereby strengthening the validity of the meta-analysis for this outcome. Nevertheless, it is always important to consider the clinical and methodological context, as well as the number and size of the studies, since a small dataset may limit the ability to detect true heterogeneity.

### 3.7. Assessment of Publication Bias

Egger’s test was used to detect potential publication bias in the meta-analyses performed. This statistical test evaluates asymmetry in funnel plots and, therefore, the possibility that studies with negative or non-significant results may not have been published. A *p*-value < 0.05 suggests the presence of publication bias.

The results were as follows: AOFAS: *p* = 0.2996; VAS: *p* = 0.0005; Dorsiflexion (FDTPA): *p* = 0.5029.

Accordingly, the results for the AOFAS scale and dorsiflexion range were considered free of publication bias in favor of improvement in the treatment group. However, for the VAS, the presence of potential publication bias was noted.

**Figure 5:**
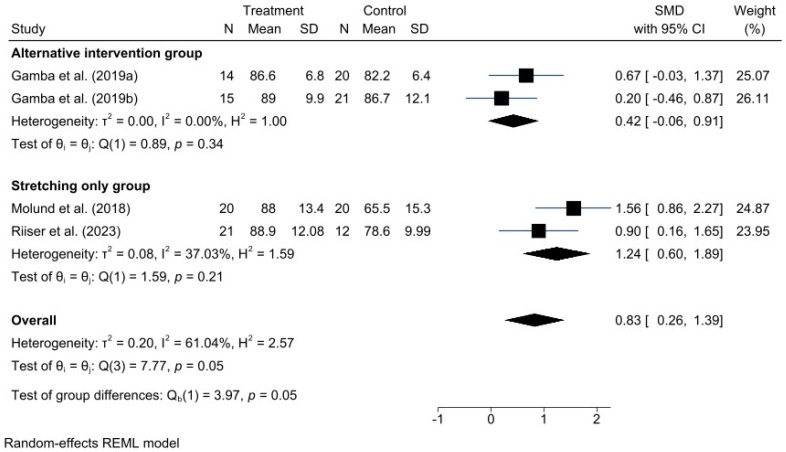
Forest plot of subgroups by type of intervention in AOFAS. Studies included: Gamba et al., 2019a [16]; Gamba et al., 2019b [27]; Molund et al., 2018 [12]; Riiser et al., 2023 [15].

**Figure 6:**
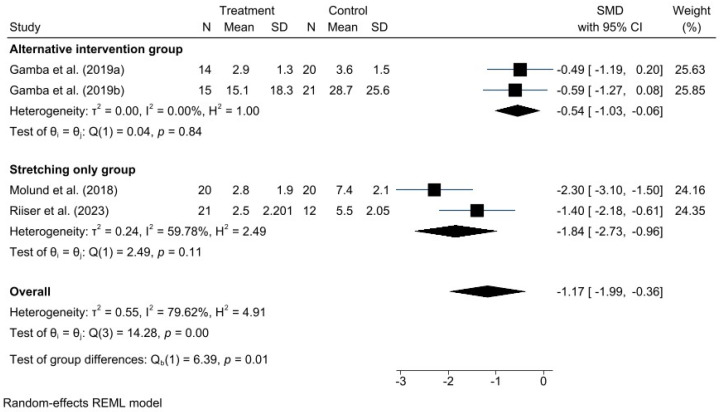
Forest plot of subgroups by type of intervention in VAS. Gamba et al., 2019a [16]; Gamba et al., 2019b [27]; Molund et al., 2018 [12]; Riiser et al., 2023 [15].

Given the high heterogeneity represented by the I^2^ values in the forest plots, different potential sources of heterogeneity were explored. Subgroup analyses were conducted to examine causes such as variations in control group interventions and differences in postoperative follow-up duration, based on AOFAS and VAS scores. These subgroup results are presented in the following additional forest plot graphs (Figure 5 and Figure 6).

The subgroup analysis in this case differentiates studies comparing GR with OPF from those comparing GR with stretching exercises. Therefore, the different types of control group interventions can be considered a potential source of the observed heterogeneity.

**Figure 7:**
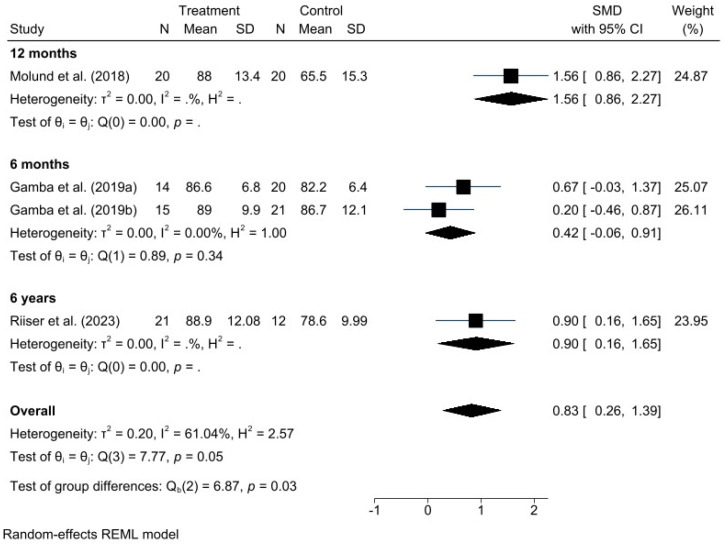
Forest plot of subgroups by postoperative time in AOFAS. Gamba et al., 2019a [16]; Gamba et al., 2019b [27]; Molund et al., 2018 [12]; Riiser et al., 2023 [15].

**Figure 8:**
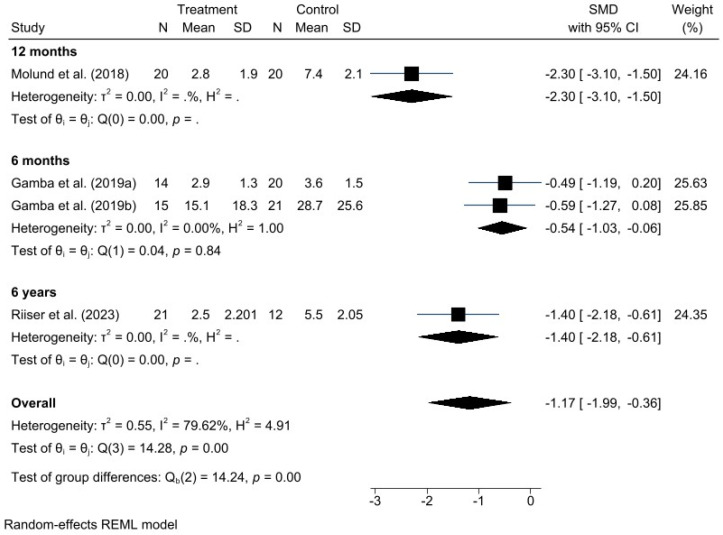
Forest plot of subgroups by postoperative follow-up time in VAS. Gamba et al., 2019a [16]; Gamba et al., 2019b [27]; Molund et al., 2018 [12]; Riiser et al., 2023 [15].

Postoperative outcomes were assessed at different follow-up times across the included studies, ranging from six months to six years. For this reason, follow-up duration is also considered a potential source of heterogeneity (Figure 7 and Figure 8).

## 4. Discussion

The findings of this meta-analysis indicate that gastrocnemius recession is associated with clinically meaningful improvements in foot function, pain, and ankle dorsiflexion in patients with recalcitrant plantar fasciitis, particularly in those presenting with isolated gastrocnemius contracture [8,12,14].

### 4.1. Clinical Interpretation of Functional and Pain Improvement

The improvements in function and pain observed after gastrocnemius recession are consistent with the hypothesis that correction of functional equinus reduces tensile stress transmitted to the plantar fascia and enhances load distribution during gait. This pattern aligns with previous randomized trials, in which surgically treated patients demonstrated superior clinical outcomes compared with stretching-only or prolonged conservative management [12,15].

When compared with plantar fasciotomy, the included studies suggest that although both procedures may achieve symptomatic relief, gastrocnemius recession offers a more conservative clinical profile by preserving fascial integrity and potentially reducing the risk of medial arch alteration and load redistribution across the midfoot and lateral column [16,27]. This distinction appears especially relevant in active patients or in those at risk of midfoot collapse, as described in studies reporting complications following extensive fascial release [27,31].

Clinical outcomes following gastrocnemius recession may also be influenced by pre-existing structural deformities. Recent evidence suggests a significant correlation between hindfoot valgus and first ray insufficiency, which are often co-existing factors in patients with altered foot mechanics. Addressing these associated conditions might be crucial for improving the success rate in recalcitrant cases [32].

### 4.2. Comparison with the Literature and Sources of Discrepancy

Overall, the results of the present meta-analysis are consistent with previous evidence reporting sustained reductions in pain and improved function following proximal gastrocnemius recession, with high patient satisfaction and a low rate of postoperative complications [12,15,16,33,34,35]. However, the magnitude of clinical benefit varies among studies, likely reflecting differences in:

(a) Surgical technique (proximal vs. distal approaches);
(b) Patient selection criteria (severity of contracture, symptom duration);
(c) Length of postoperative follow-up [8,23,24,35].

These variations highlight the need to interpret outcomes within an individualized clinical framework, noting that the benefits appear greater in patients with objectively documented gastrocnemius contracture and failure of conservative treatment, as reported in both short- and long-term follow-ups by Molund and Riiser [12,15].

### 4.3. Biomechanical Considerations and the Role of Postoperative Management

The trials included in this review describe postoperative strategies based on early mobilization and progressive stretching of the triceps surae, which may help consolidate functional recovery by facilitating adaptation of load patterns without directly compromising plantar fascial integrity [12,16,27]. From a biomechanical standpoint, this approach contrasts with plantar fasciotomy, where loss of the windlass mechanism, lateral column overload, and medial arch collapse have been reported when fascial release is excessive [9,27]. In this context, gastrocnemius recession may be understood as a procedure that targets the origin of fascial overload while preserving the structural architecture of the fascia [8,14,31].

### 4.4. Clinical Implications

Taken together, the available evidence supports gastrocnemius recession as an appropriate surgical option for patients with recalcitrant plantar fasciitis, isolated gastrocnemius contracture, and documented failure of conservative therapy, particularly in scenarios where preservation of plantar fascial integrity and midfoot stability is a therapeutic priority [8,12,15,16,31]. Nevertheless, surgical decision-making should be grounded in a comprehensive clinical assessment that includes biomechanical evaluation, functional examination, and patient expectations.

### 4.5. Limitations and Future Directions

The interpretation of these findings requires caution due to methodological heterogeneity among studies, small sample sizes, and the potential presence of publication bias in pain outcomes. However, the specific evidence of publication bias in VAS pain outcomes (*p* = 0.0005) suggests that the observed magnitude of symptomatic improvement should be interpreted with caution, as studies reporting less favorable or null effects may be underrepresented in the available literature. These constraints are consistent with limitations previously identified in the surgical equinus and gastrocnemius recession literature [8,23,31]. To strengthen the current body of evidence, future research should prioritize multicenter randomized trials with larger cohorts, direct comparisons between recession techniques (e.g., Barouk, Strayer, Baumann), stratification by contracture severity, and inclusion of objective biomechanical measures such as plantar pressure analysis and gait assessment [8,36,37,38].

## 5. Conclusions

GR emerges as an effective and safe therapeutic option for the treatment of recalcitrant plantar fasciitis, particularly in patients who have not adequately responded to conservative interventions. This systematic review and meta-analysis demonstrate that GR yields clinically significant benefits in three key domains: improvement in foot function (measured by the AOFAS scale), reduction in pain (assessed using the VAS), and increased ankle dorsiflexion range (FDTPA). These positive effects were sustained even in studies with extended follow-up, suggesting the durability of the surgical outcome over time. Moreover, the results indicate that GR offers advantages over other surgical techniques, such as plantar fasciotomy, not only in terms of clinical efficacy but also by enabling faster recovery, lower postoperative morbidity, and preservation of fascial architecture. Additionally, its implementation via minimally invasive or ultrasound-guided approaches further enhances the safety profile of the procedure.

## Figures and Tables

**Table 1:** Comparative Overview of Surgical Techniques for Gastrocnemius Recession.

Origin and Context	Silfverskiöld (1924); Barouk (1980)	Baumann & Koch (1989)	Vulpius (1913); Strayer (1950); Baker (1956)	Dr. Michael Hoke (1931)
Principles and Applications	Silfverskiöld: releases both heads of the gastrocnemius. Barouk: cuts the gastrocnemius aponeurosis at the junction between the muscle and aponeurosis, focusing on the medial head.	Lengthens only the gastrocnemius, fully preserving the soleus.	Strayer: cuts the gastrocnemius tendon at the musculotendinous junction, leaving the soleus intact. Vulpius: lengthens at the junction between gastrocnemius and soleus. Baker: cuts the conjoint aponeurosis.	Two medial incisions and one lateral incision.
Advantages	Silfverskiöld/Barouk: Less invasive compared to distal techniques. Barouk’s approach has evolved into ultrasound-guided/percutaneous techniques that minimize scarring.	The evolution toward ultrasound-guided techniques has further reduced tissue trauma and improved recovery.	These three techniques provide greater correction of equinus compared to more proximal approaches.	Greater correction in severe deformities. Percutaneous procedure with minimal scarring.
Disadvantages	May be insufficient for very severe contractures.	Less lengthening compared to distal techniques.	Strayer/Vulpius/Baker: Higher risk of complications: sural nerve injury, overlengthening, more visible scarring.	Risk of overlengthening if not properly calibrated.

**Table 2:** Description of Studies: Population, Interventions, Outcomes, and Key Findings in Surgical Treatments for Plantar Fasciitis.

Study	Treated Condition	Surgical Intervention	Control/Comparison Group	Number of Patients/Extremities	Follow-Up	Key Results	Main Conclusions
Molund et al., 2018 [12]	Chronic heel pain (plantar fasciitis) with isolated gastrocnemius contracture (IGC)	Medial gastrocnemius proximal recession (PMGR) + stretching exercises	Stretching exercises only	40 patients (20 operative group, 20 non-operative group)	12 months	The operative group had significantly better scores on AOFAS (59.5 to 88.0), pain (VAS), and SF-36 at 12 months (*p* < 0.05). Ankle dorsiflexion in the operative group increased from 6 to 10.5 degrees. No differences were observed between groups in Achilles tendon function	Medial gastrocnemius proximal recession with a stretching program was a safe and effective method for treating chronic heel pain.
Gamba et al., 2019 [16]	Recalcitrant plantar fasciitis (RPF)	Gastrocnemius release (LG) at the popliteal fossa	Open partial plantar fasciotomy (OPF)	34 patients (14 LG, 20 OPF)	6 months	Both groups showed statistically significant improvement in EVA and AOFAS scores. The LG group showed faster postoperative improvement, visible by the first month. No significant differences were found between the two groups at the end of follow-up in terms of function (AOFAS, *p* = 0.36), pain (EVA, *p* = 0.1), satisfaction (*p* = 0.61), or health perception (SF-36)	Both LG and OPF are effective surgical treatments for RPF. LG offers a faster postoperative recovery for patients.
Gamba et al., 2019 [27]	Recalcitrant plantar fasciitis	Proximal medial gastrocnemius release	Open plantar fasciotomy	36 patients (15 PMGR, 21 OPF)	Up to 1 year	Both procedures were effective. No significant differences at 1 year in AOFAS (*p* = 0.24), VAS (*p* = 0.14), or any item of SF-36. Overall satisfaction was very good in both groups (85.8% PMGR, 89.5% OPF). Faster recovery was observed in the PMGR group. No calf strength loss was observed.	PMGR and OPF are effective and safe surgical options for RPF patients. Although there was no superior technique, the authors consider PMGR the preferred technique to avoid possible biomechanical complications related to OPF.
Riiser et al., 2023 [15]	Chronic plantar fasciitis combined with isolated gastrocnemius contracture (IGC)	Medial gastrocnemius proximal recession (PMGR) + stretching exercises	Stretching exercises only	40 initial patients (33 completed 6-year follow-up; 7 crossovers)	6 years	The operative group showed significantly better results at 6 years in AOFAS (88.9 vs. 78.6, *p* = 0.012), pain measured by VAS (2.5 vs. 5.5, *p* < 0.001), and total MOxFQ score (24.4 vs. 45.9, *p* = 0.05). No differences between groups in ankle dorsiflexion or Achilles complex performance at 6 years. The effect of surgery did not diminish after 6 years.	The results show that improved function and reduced pain from PMGR and stretching are better compared to stretching alone after 6 years of follow-up. PMGR is considered a safe procedure with good long-term outcomes.

The gastrocnemius recession was originally described by Strayer (1950) [25] as a distal recession. The proximal medial variant (PMGR) [31] used in the included studies was subsequently developed and popularized by Pierre Barouk, and the term LG (gastrocnemius release) functionally refers to the same family of proximal medial gastrocnemius release procedures [31].

**Table 3:** ROB-2 (Risk Of Bias In Randomized Trials).

Author/Year	It. 1	It. 2	It. 3	It. 4	It. 5	Overall
Molund et al., 2018 [12]	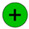	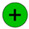	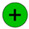	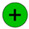	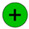	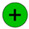
Riiser et al., 2023 [15]	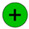	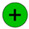	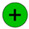	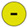	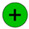	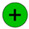
Gamba et al., 2019a [16]	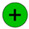	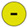	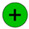	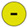	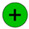	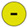
Gamba et al., 2019b [27]	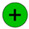	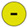	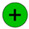	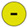	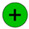	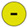

Note: it. 1 = Bias due to randomization—Was the randomization process conducted appropriately to ensure comparability between groups? it. 2 = Bias due to deviations from Intended Interventions—Were there deviations from the assigned intervention that could affect the study’s validity? it. 3 = Bias due to Missing Data—Was Missing Data handled appropriately, or could its absence introduce bias in the results? it. 4 = Bias in Measurement of Outcomes—Were outcome assessments conducted in a way that minimized measurement bias? it. 5 = Bias in Selection of Reported Re-sults—Was there selective reporting of results, leading to potential misrepresentation of findings? Overall = Overall Risk of Bias—Final assessment considering the cumulative effect of all bias do-mains. Interpretation: “Low” risk of bias was represented by the color green, indicating minimal concerns regarding the study’s validity, while the color yellow represented “some concerns”.

**Table 4:** Summary of Evidence Quality Assessment Using the GRADE Approach.

Study	Risk of Bias	Inconsistency	Indirectness	Imprecision	Publication Bias	Overall Quality
Molund et al., 2018 [12]	Low	Not reported	No	No	No	High
Gamba et al., 2019a [16]	Moderate	No	No	Moderate	No	Moderate
Gamba et al., 2019b [27]	Moderate	No	No	No	No	Moderate
Riiser et al., 2023 [15]	Low	Not reported	No	Mild	No	High

## Data Availability

Data are contained within this article.

## Supplementary Materials

The following supporting information can be downloaded at: https://www.mdpi.com/article/10.3390/jcm15020616/s1, Supplementary File S1: PRISMA 2020 checklist.

## Abbreviations

The following abbreviations are used in this manuscript:

AOFAS	American Orthopaedic Foot and Ankle Society
FDTPA	Foot Dorsiflexion Test with Knee in Extension
FAAM	Foot and Ankle Ability Measure
OPF	Open Plantar Fasciotomy
FFI	Foot Function Index
RPF	Recalcitrant Plantar Fasciitis
GR	Gastrocnemius Recession
GRADE	Grading of Recommendations, Assessment, Development and Evaluation
CI 95%	95% Confidence Interval
MOxFQ	Manchester Oxford Foot Questionnaire
PMGR	Proximal Medial Gastrocnemius Recession
PROSPERO	International Prospective Register of Systematic Reviews
PRISMA:	Preferred Reporting Items for Systematic Reviews and Meta-Analyses
RoB 2	Risk of Bias 2 (Cochrane risk of bias tool for randomized trials)
SF-36	Short Form Health Survey (36 items)
SMD	Standardized Mean Difference
VAS	Visual Analog Scale

## Appendix A

### Appendix A.1. Searches Strings for Electronic Databases

The original search string was created in PubMed. The searches strings employed for Web of Science and MEDLINE via PubMed were translated using an automatic online tool www.sr-accelerator.com/Polyglot (accessed on 10 May 2025):

#### Appendix A.1.1. Search Strategy for PubMed

URL: https://pubmed.ncbi.nlm.nih.gov (accessed on 10 May 2025)

(“gastrocnemius recession” OR “gastrocnemius release” OR “gastrocnemius lengthening” OR “gastrocnemius slide” OR “Strayer procedure”) AND (“plantar fasciitis” OR “plantar heel pain” OR “heel spur syndrome” OR “chronic plantar fasciopathy”).

#### Appendix A.1.2. Search Strategy for Web of Science

URL: https://www.webofscience.com/wos/alldb/basic-search (accessed on 10 May 2025)

Filters: no filters were applied.

“plantar fasciitis” AND “gastrocnemius recession”.

#### Appendix A.1.3. Search Strategy for Scopus

(“gastrocnemius recession” OR “gastrocnemius release” OR “gastrocnemius lengthening” OR “gastrocnemius slide” OR “Strayer procedure”) AND (“plantar fasciitis” OR “plantar heel pain” OR “heel spur syndrome” OR “chronic plantar fasciopathy”).
